# Crossmodal Language Comprehension—Psycholinguistic Insights and Computational Approaches

**DOI:** 10.3389/fnbot.2020.00002

**Published:** 2020-01-31

**Authors:** Özge Alaçam, Xingshan Li, Wolfgang Menzel, Tobias Staron

**Affiliations:** ^1^Natural Language Systems Group, Department of Informatics, University of Hamburg, Hamburg, Germany; ^2^Reading and Visual Cognition Lab, Institute of Psychology, Chinese Academy of Science, Beijing, China

**Keywords:** language comprehension, crossmodality, psycholinguistics, incrementality, prediction, speaker intention

## Abstract

Crossmodal interaction in situated language comprehension is important for effective and efficient communication. The relationship between linguistic and visual stimuli provides mutual benefit: While vision contributes, for instance, information to improve language understanding, language in turn plays a role in driving the focus of attention in the visual environment. However, language and vision are two different representational modalities, which accommodate different aspects and granularities of conceptualizations. To integrate them into a single, coherent system solution is still a challenge, which could profit from inspiration by human crossmodal processing. Based on fundamental psycholinguistic insights into the nature of situated language comprehension, we derive a set of performance characteristics facilitating the robustness of language understanding, such as crossmodal reference resolution, attention guidance, or predictive processing. Artificial systems for language comprehension should meet these characteristics in order to be able to perform in a natural and smooth manner. We discuss how empirical findings on the crossmodal support of language comprehension in humans can be applied in computational solutions for situated language comprehension and how they can help to mitigate the shortcomings of current approaches.

## 1. Introduction

Enabling artificial systems to engage in a natural and smooth spoken dialog with humans is a major scientific and technological challenge. To make this dream come true, developers have always sought inspiration from the only model available, the human. Compared to other means of communication, the expressiveness and flexibility of natural language to accommodate to vastly changing application needs is unparalleled. A closer look at the phenomenon shows that the language faculty is not an isolated capability of human cognition. Instead, it maintains close ties to other cognitive subsystems, like visual perception, from where it receives information about the surrounding environment and into which it feeds back. While the additional extra-linguistic information by and large provides an instrumental contribution to overcome comprehension difficulties, for instance in the areas of ambiguity or reference resolution, the linguistically conveyed information drives the attention of the listener toward the relevant areas of the visual stimulus that can maximize the information gain.

Such a closed feedback loop can be particularly productive if the relevant comprehension results are available early enough, so that they can exert their influence on the visual system. Only then can the visual percepts improve the ongoing linguistic processing by means of the specific information they contribute. Obviously, language comprehension and visual perception work together for common benefit in a closely time-locked manner, producing tentative results. Language comprehension not only amounts to a kind of understanding of what has been said, but also has to determine as early as possible the reference of linguistic expressions to entities in the world as well as the relationships these entities maintain among each other.

From a technical perspective, the use of visual cues for improving natural language processing can be studied as a problem of information fusion (Bloch, 2008). In contrast to, for instance, combining spatial information from a range of different cameras or laser range finders, here the integration has to happen on a conceptual level, because vision and speech do not usually share a common metrical space beyond the task of sound source localization. Linguistically described concepts and visually perceived entities have to be mapped by means of an abstract representation that allows the listener to achieve a coherent interpretation of the current state of affairs in spite of partially deviating contributions.

Consequently, the fusion metaphor of combining the output of two independent information sources will not be viable if we aim for the more ambitious goal of taking advantage of a closed feedback loop between language and vision. Both subsystems seem to be developed into separate components that are able to produce and receive contributions from one another while they are processing the input; hence, they interact with each other. This situation raises many questions on how the human mind organizes this interplay in detail and how certain aspects of it can be implemented in an artificial agent, thereby leading to systems that, rather than fuse both modalities, maintain separate, but interacting representations.

It is this interactive nature of the cooperation between two complementary modalities that we are mostly concerned with. We not only study language comprehension that is sensitive to the visual information from a task-oriented spatially embedded scenario, but also considers the guidance language comprehension can provide to drive the hearer's attention to the most relevant aspects of the visual scene which might contain more detailed information vital for the ongoing process of language comprehension.

We are mainly concerned with the mechanisms of meaning recovery, ambiguity resolution and visual grounding, focusing specifically on the syntactic and semantic processes at the lexical and sentential level. The impact of emotion, irony, metaphoric use etc. is not considered. Speaker-related information is reduced to the bare utterance she produced, ignoring any cues such as lip movements or gestures. We also do not cover problems or computational solutions for language generation, speech recognition, and visual perception. Visual stimuli are assumed to be static ones but subject to a kind of attention processing where visual comprehension also evolves over time.

To better understand the underlying mechanisms of such a highly complex behavior, we identify a range of performance characteristics that seem to contribute crucially to a generally highly successful and efficient processing architecture. Nevertheless, we set out to analyze these performance characteristics in a holistic way that sheds light into their intertwined nature. We also interpret them as challenges for computational systems designed to be capable of engaging themselves in a task-oriented dialog with a human interlocutor. To this end, we review important findings from psycholinguistic research and confront them with recent advances in building crossmodal natural language comprehension systems, trying to identify potential drawbacks of existing computational solutions and to learn from the human model to overcome them.

We adopt a fairly broad perspective on the language capabilities of artificial systems, which transcends limited command-and-control approaches. Instead of dealing with narrow-domain approaches, we envision a kind of mixed-initiative system that is capable of sharing information, discussing alternative options and negotiating action strategies toward a common goal with its human partner. The linguistic means, required to achieve such a level of communicative competence are shortly outlined in section 2. In subsequent sections we discuss how the visual input can help to resolve linguistic ambiguities (section 3), how the mapping between visually perceived entities and their linguistic descriptions can be established (section 4), how language can drive visual attention and support visual search (section 5), and how the two modalities can be combined to reach a maximum degree of synergy (section 6). We then turn to the temporal aspects of the interaction between language and vision, concluding that such a benefit can only be achieved if the mutual contributions are available early enough (section 7), possibly even before they actually are available in the discourse (section 8). Finally, we discuss some heuristics humans apply to speed up language comprehension (section 9).

## 2. Speaker Intention

In a task-oriented setting, it is of particular importance to determine the intention of the speaker, i.e., what she wants the listener to do: accept a message, answer an information request, carry out an action, etc. If visual information is involved in this process, it will be expected to contribute to successfully accomplishing this task.

To achieve feasible solutions, language-based human-machine communication traditionally restricts the interaction to only explicit commands that the machine is meant to comply with. In such a case, identifying the intention of the speaker amounts to

- selecting the desired action from those the machine can carry out (c.f. [1] in the example below), and
- unambiguously determining the referential objects involved in the action by means of additionally given information about their types [2], properties [3], and (spatial) relationships [4]:

“*Bring*_[1]_ *me the blue*_[3]_ *mug*_[2]_ *from the table*_[4]_*.”*

As shown by Gorniak and Roy (2005), automatic reference resolution in situated language processing benefits from taking speaker intention into account. Their system follows the instructions of the user in a role playing video game. To determine her intention, a probabilistic parser for context free grammars predicts which objects from the environment the user will talk about next. These objects, together with the actions that seem most likely in the current state of the user's plan, are interpreted as intention. The authors reported that combining language and vision with the intention of the speaker yielded the best reference resolution results (see Table 1). The tasks of intention detection, reference resolution, crossmodal information fusion and prediction are closely tied to each other rather than addressed separately by the system.

**Table 1:** The number of correctly resolved referents given different combinations of information sources in the role playing video game setting of Gorniak and Roy (2005).

**Language + Vision**	**Language + Intention**	**Language + Vision + Intention**
27/90 (30.0%)	21/90 (23.0%)	**50/90 (56.0%)**

*Here and in all other tables, numbers in bold indicate the results with the highest accuracy for each approach.*.

**Figure 1:**
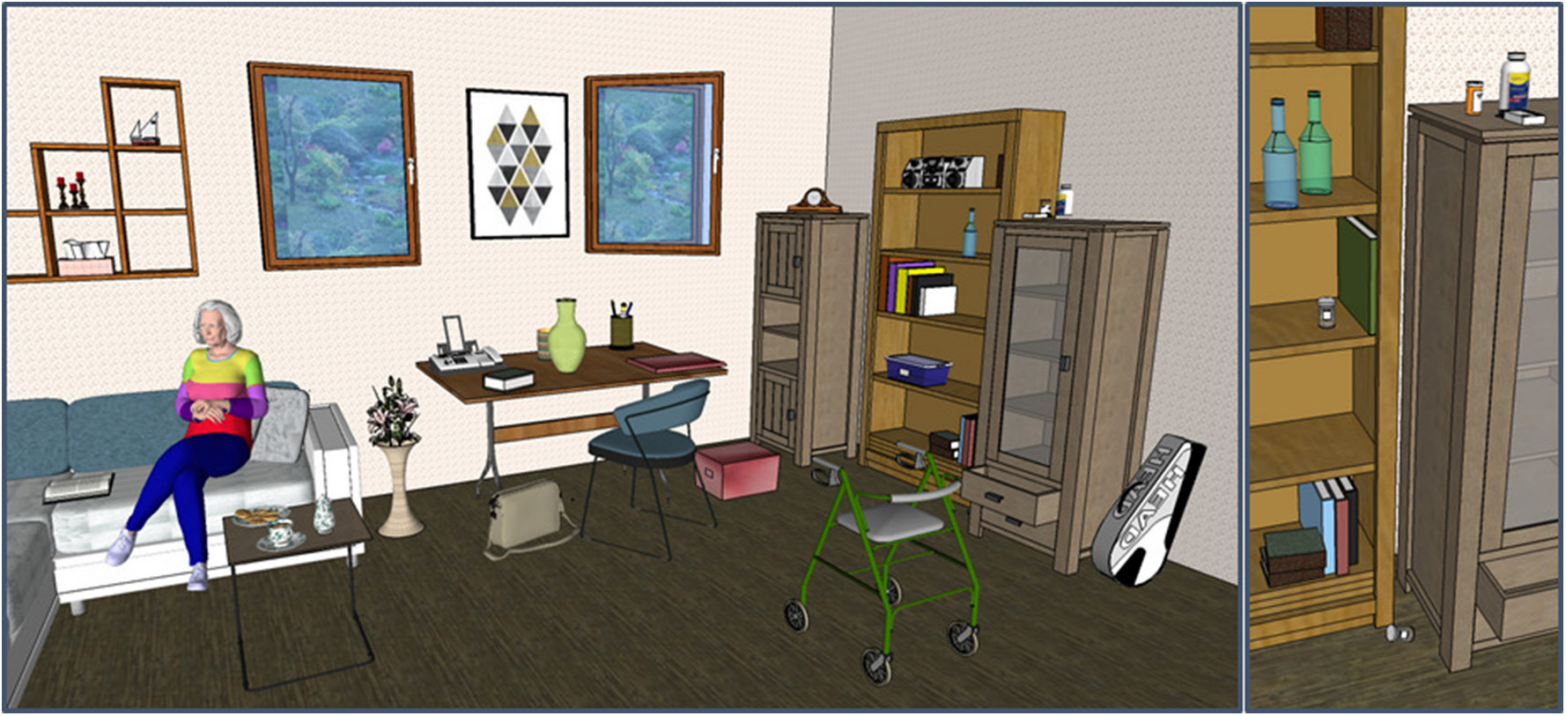
An example image for a living room scenario.

While the system of Gorniak and Roy (2005) is limited to command execution, the task becomes considerably more difficult in the case of collaborative problem solving, where dynamic effort from both parties is required. Collaborative problem solving usually happens in structurally rich visual environments like the one in Figure 1, which contains several windows, cabinets, boxes, pills, magazines, bottles etc., some of them even (partially) occluded from the viewer's perspective. Objects in such an environment can be referred to in quite different, sometimes underspecified manners, and their sheer number naturally creates a vastly larger space for reference resolution. Under these conditions, the optimal interplay between language, visual information and world knowledge is crucial.

Collaborative problem solving also requires the negotiation of common goals and a solution strategy to achieve them despite unexpected difficulties that may arise during problem solving. Thus, the intention of the speaker can no longer be restricted to the special case of giving commands, but has to be inferred from her utterance. Often, information needs to be requested and exchanged, for instance by means of direct or indirect inquiries, which both may come in various forms, such as:

**Table:** 

direct inquiry as a direct question	“*How many pills are left?”*
indirect inquiry as a direct question	“*Did you count the pills?”*
direct inquiry as an indirect question	“*Do you know, whether we have enough pills?”*
indirect inquiry as an imperative	“*Please count the leftover pills.”*
direct inquiry as a confirmation question	“*The bottle is really empty?”*

and assertions about:

**Table:** 

the current state of affairs	“*The book lies on the couch.”*
an embedded current state of affairs	“*You can find the book on the couch.”*
an embedded previous state of affairs	“*I left the book on the couch.”*

or even embedded states of affairs expressed, for instance, as:

**Table:** 

a confirmation question	“*The book is no longer lying on the couch?”*

Negotiating a joint solution strategy for a given problem also requires means for establishing and maintaining consensus, for instance, making:

**Table:** 

a direct proposal	“*To pack the bottles, we need a bigger box.”*
a counterproposal	“*It might be better to first check the pills.”*
or an indirect counterproposal	“*This box is bigger.”*

establishing consent signaling:

**Table:** 

weak agreement	“*If you think so.”*
or strong agreement	“*That's a great idea.”*

or rejecting a proposal:

**Table:** 

indirectly	“*The book is too boring.”*
or with an explanation	“*No, I am tired.”*

All these different kinds of communicative goals can be expressed by a very limited set of general utterance types, namely declarative (direct or indirect), interrogative and imperative sentences, which actually can be spelled out by means of an extremely rich inventory of syntactic variation. The same kind of sentence type or syntactic pattern can be used to express quite different intentions. Thus, determining the correct intention is not always straightforward.

Moreover, the hearer will be faced not only with a much broader spectrum of possible intentions and syntactic variation, but also with indirect utterances where the real intention (illocution) is hidden. Implicit commands like “*Have you seen my book?”* or “*I left the book on the table.”* or “*I'd like to read.”* require the hearer to reconstruct the underlying intention (“*Bring the book here.”*) (Clark et al., 1983; Kelleher and Costello, 2009; Gundel et al., 2012). Expressing this intention explicitly most often results in unwieldy utterances, whereas leaving part of it underspecified contributes substantially to the ease and economy of language communication. Reconstructing the intended purpose requires more or less complex inferences that rely on the available information about the immediate environment and the world in general.

On the other hand, overspecification is also frequent in natural language communication. Speakers usually use it when one of the properties of the target entity is salient but has no contrastive value (Engelhardt et al., 2006; Koolen et al., 2011; Rubio-Fernández, 2016). From the perspective of language comprehension, such a redundancy poses no serious problem, unless it creates an inconsistency that needs to be resolved. Even though the necessity to deal with unnecessarily long expressions could affect the response time, the additional processing effort may be compensated for by faster reference resolution in complex environments.

## 3. Resolution of Linguistic Ambiguities

One of the most prevalent difficulties in language comprehension is the number of ambiguities inherent in both lexical items and complex structures. Therefore, developing algorithmic approaches for disambiguation has always been a major concern when designing natural language understanding systems. These systems usually rest on combinatorial decision procedures combined with a powerful scoring mechanism as the basis for preferential reasoning. However, in restricted domains, the number of linguistic expressions with several completely different meanings is fairly low. In the living room scenario (see Figure 1), the alternative readings of polysemous words like *chair* or *window* can be easily excluded from consideration. Part-of-speech ambiguities of words like *open* (adjective vs. verb) or *book* (noun vs. verb) are more relevant in such a scenario. They may create spurious interpretations in the comprehension process and thus inflate the space of possible intermediate hypotheses. Similar processing problems are created by truly structural ambiguities like the famous case of prepositional phrase attachment (“…*the lid of the box on the table.”*). A fourth type of ambiguities arises from language-internal references, which can be established for example by means of different pronouns or definite noun phrases (“…*but it is broken.”*).

For all kinds of ambiguous constructions, crossmodal evidence may help to resolve the ambiguity by either re-ranking the possible interpretations or even excluding some of them according to their plausibility in the visual world. Especially when linguistic cues alone do not suffice to determine the actually intended interpretation, for instance because it contradicts both frequency of use and human preferences, crossmodal interaction will become indispensable to achieve an effective and timely disambiguation.

In general, ambiguities can be dealt with most efficiently if they are resolved locally. Otherwise, their combinatorics will overwhelm the comprehension system. Therefore, it is important to have the disambiguating information available early enough. Visually contributed information does exactly this: In many cases, it can be extracted from the visual environment long before it is actually needed. The linguistic channel, in contrast, provides its information sequentially. Thus, the comprehension system always needs to wait until the relevant contributions appear in the ongoing utterance. This may cause serious comprehension problems and processing delays.

Although humans use visual information for resolving structural ambiguities, they seem to acquire this ability at a fairly late stage in their linguistic development. While, by the age of five, children are already able to apply bottom-up lexical information supplied by the verb to correctly attach a prepositional phrase (Spivey-Knowlton and Sedivy, 1995), incorporating extra-linguistic top-down knowledge required to deal with long-range dependencies comes later (Atkinson et al., 2018). Obviously, the optimal combination of visual and linguistic cues is a capability that can and needs to be developed, reinforced by positive feedback.

The facilitating role of visual information has been demonstrated by Tanenhaus et al. (1995) using a task of incremental thematic role assignment. In their seminal study, participants were given sentences with a prepositional phrase (PP) attachment ambiguity, where different semantic interpretations are possible depending on how the linguistically encoded entities are assigned to the different thematic roles of the verb. In the example sentence, “*Put the apple on the towel in the box.”*, the PP *on the towel* can be interpreted as the goal point of the movement action in

“*Put [the apple]*_Theme_ *[on the towel [in the box]*_Location_*]*_Goal_”

or as a modifier of an apple (namely the location of the apple)

“*Put [the apple [on the towel]*_Location_*]*_Theme_ *[in the box]*_Goal_”

In the absence of visual information, both interpretations are possible, but linguistic attachment preferences will assign *towel* as a goal of the putting event immediately after hearing the PP *on the towel*. Then, after being exposed to the next PP *in the box*, re-evaluation of the already assigned thematic role from Goal to a Location role is required, and the *box* becomes the goal. If, on the other hand, the listener has access to a picture that shows a towel in a box, early reference resolution will happen without any need to revise the initial hypothesis.

Baumgärtner (2013) studied the problem of visually guided ambiguity resolution using an incremental, crossmodal parser. He was able to show that crossmodal interaction of language and vision indeed helps to resolve global as well as temporal ambiguities that were truly ambiguous without the contribution of the visual channel. He applies a broad-coverage, grammar-based syntactic parser for German extended by a component for thematic role assignment (McCrae and Menzel, 2007; McCrae, 2009; Beuck et al., 2013). The grammar is encoded by means of weighted constraints that license linguistically meaningful structures and provide for preferential reasoning capabilities even in case of conflicting linguistic preferences. Sentences are processed on a word-by-word basis, and partial analyses are extended and re-evaluated after each new word. Predictions are modeled explicitly by means of placeholders which can be incorporated into the analysis if this leads to a solution with a higher plausibility score based on linguistic well-formedness criteria.

The visual information was made available by Baumgärtner (2013) in the form of manual annotations of the most relevant relationships that could in principle be extracted from a picture. The mapping between linguistically and visually expressed entities, i.e., the crossmodal reference resolution, is achieved by means of additional constraints for linguistic structures that also have access to the visual input. Hence, language and vision interact in a bidirectional manner through the normal constraint solving mechanism, and visual information guides the parsing process. In turn, the intermediate parsing results guide the visual attention. The predictions are combined with low-level indicators of visual saliency, like saturation and contrast (Itti and Koch, 2000), to create a modified saliency landscape that correctly predicts the eye fixations of human subjects. If the visual channel is missing, parsing will be performed based on the linguistic input alone.

Since the constraints that establish the mapping between the two modalities are weighted, the mutual influence is based on preferences rather than hard consistency requirements, and the bias between the input channels can be adjusted, thereby reducing the influence of noisy and potentially contradicting input from the visual channel.

Baumgärtner (2013) uses the placeholders as referents for concepts that participate in a visually depicted action with a specific thematic role, and therefore are likely to occur with this role in the remainder of the sentence. These results indicate that, similar to the experiments on intention recognition, a system architecture that combines ambiguity resolution with reference resolution, crossmodal information fusion and prediction is able to deal with ambiguity more effectively and more rapidly, thus making the system more responsive.

Linguistic stimuli with (temporal or global) structural ambiguities are used intensively in psycholinguistic research, as they open an excellent observation window into the hidden processes of human language comprehension. They provide valuable insights into the time course of language comprehension in general and ambiguity resolution in particular, for example in combination with eye-tracking analyses. The relative amount of eye fixations on the different parts of a static visual environment is interpreted as a signal at which point in time reference has been successfully established, and what kind of information was required to achieve this. This approach has been named the visual world paradigm; see Huettig et al. (2011) for a review.

## 4. Crossmodal Reference Resolution

Crossmodal reference resolution can be understood as another kind of ambiguity resolution. It does not concern the reference to linguistically described entities of the world, but to those that can be inferred from the visual stimulus. In rich environments, a lexical expression can possibly refer to a number of entities: For instance, there are multiple books in the scenario depicted in Figure 1. Also, expressions can be related to entities in the environment that might be confused with other objects of similar appearance. Moreover, visual objects sometimes are partly occluded from the perspective of the listener, or are otherwise difficult to perceive. Again, the resulting combinatorics can be controlled best if the space of possible mappings can be constrained as early as possible. This creates a very strong incentive for an incremental (and predictive) processing mode, which facilitates the interaction between the two modalities as early as possible in order to exchange disambiguating information in both directions.

Even under ideal conditions, there is no exact mapping between the concepts contributed linguistically and the visually perceived information. The two modalities differ in their ability to accommodate different aspects and granularities of conceptualizations. Language, for example, is well-suited to describe entities with a very fine grained inventory of categories, which most often are difficult to distinguish visually: For example, it is possible to linguistically refer to the same entity by means of expressions like *the book, the paperback, the thriller*, or *the Agatha Christie*. The large degree of linguistic variability that is available to refer to entities is not limited to objects, but also extends to actions that can be lexicalized in quite different ways, such as *to talk, to discuss, to negotiate, to chat*, etc. The visual channel, on the other hand, is usually superior when spatial properties are involved. Incorporating the information from the visual channel, the reference for a relative expression like *the mug on the left* can easily be determined.

Generally, a simple combination of linguistic and visual information will not suffice to establish the mapping between the linguistic concepts and their visual correspondences. At least some kind of ontological knowledge will be indispensable to solve this problem satisfactorily (McCrae, 2009). Often, however, reference resolution will require considerably more complex inferences: For instance, the sentence “*Bring me my grandma's book.”* with respect to Figure 1 requires either a substantial amount of background knowledge about the relatives of the speaker and their visual appearance or even less reliable non-monotonic reasoning based on the absence of other people that could be used as referential entities. Moreover, the linguistically expressed ownership relation adds uncertainty because it cannot easily be derived from a visual stimulus in general.

From a neurophysiological perspective, the mapping of (situated) language to conceptual categories is facilitated by a common brain area, namely the hippocampal structure (Duff and Brown-Schmidt, 2012; Moscovitch et al., 2016). A recent study by Piai et al. (2016) also revealed that the mapping between the two modalities in the hippocampus is performed incrementally and enables the prediction of upcoming words.

Psycholinguistic studies into the nature of relating instances (visual entities) to the relevant conceptual category point toward a dynamic mapping process instead of a one-shot association (Altmann, 2017). It is assumed that, based on the similarity to an already existing abstract mental representation, an episodic representation for the perceived visual entity is generated incrementally based on the expectations about the incoming sentence parts and the visual event. In return, conceptual categories are updated, which results in abstract concepts that have lost the individual details of the original instance.

Concept mapping will become even more difficult if the entity referred to undergoes a change of state as a spoken utterance unfolds. Different versions of the same instance have to be created, mapped on to each other and updated according to the actions carried out. Given the simple story “*The woman chopped an onion. Then, she fried it.”*, the different states of the onion (1) intact and raw, (2) chopped and raw and finally (3) chopped and fried need to be maintained and bound together in order to understand the utterance (Hindy et al., 2012, 2013; Altmann, 2017). Mapping the current state of the entity to its past and possible future states has to keep track of the common features, namely the visual properties of the object, its spatial relationships with the other objects in the environment, as well as the properties of the conceptual category that it belongs to.

Kruijff et al. (2010) studied the problem of dynamically evolving worlds by means of a system for human-robot interaction, whose dialogue understanding capabilities significantly improved when visual information was taken into account. Its ability to keep track of instances whose state or semantic category changes over time allows the system to talk about entities that will cease or be transformed, such as the building materials a house is built from.

Whereas Baumgärtner (2013) used manually coded constraints to realize crossmodal reference resolution, Kitaev and Klein (2017) showed that the mapping between language and vision can be learned. Their study focuses on spatial descriptors, i.e., linguistic descriptions of relations between objects in images, and their localization. Employing a neural network based on an LSTM and pretrained word embeddings, the authors were able to demonstrate that this architecture can learn the grounding of spatial descriptors and the selection of the most plausible focus point given a set of possible target locations. The system achieved an accuracy of 62.5 % compared to 16.7 % for a randomized baseline and 85.8 % for human performance.

Besides learning the mapping from language to vision, the generation of linguistic expressions can be learned from visual entities, too. Zarrieß and Schlangen (2017b) provide an overview of different machine learning approaches to generate referential expressions from images (Lazaridou et al., 2014; Schlangen et al., 2016; Zarrieß and Schlangen, 2017a), a subtask of image captioning. In contrast to such explicit mappings, many of the systems discussed throughout this article apply end-to-end neural approaches, where the crossmodal reference resolution happens implicitly.

## 5. Visual Guidance and Search

Apart from describing or referring to visually presented entities, language can also be used to guide the attention of the listener toward a certain area of the visual environment. This is also possible in artificial systems, as demonstrated, for instance, by Baumgärtner (2013). Such a shift of attention can be triggered by different means: Simply mentioning or describing an entity will cause listeners to fixate their gaze on the possible visual referents. In familiar environments or scenarios of low complexity, this happens almost involuntarily and with a very low latency. Also, the speaker can explicitly direct the visual attention of the listener by describing relevant parts of the visual environment, for example by talking about landmarks in the vicinity of an intended referent. Hearing a sentence like ”*Next to the big table there is a white tennis bag.”*, with respect to Figure 1, the listener will already look out for tables after hearing the initial prepositional phrase. She can select the larger one among them, and possibly start moving into that direction. After receiving the color attribute *white*, her visual attention can further zoom in on white objects, which are restricted to a tennis bag only a word later.

Visual attention can be guided not only by mentioning objects in the environment explicitly, but also by means of more indirect characteristics like the affordances they offer or the current state of the environment. For instance, processing the request ”*Could you please close the window?”* in the environment of Figure 1, the listener may already start the visual search for closable objects like a window, a box, or a drawer, as soon as she perceives the verb *close*. Since there is only one open item among the possible options, the window to the right can be identified as the intended target with a very small delay. This result can be produced so rapidly only through a combination of incremental processing with crossmodal interaction and the predictions derived from the selectional restrictions of the verb.

In cases where possible candidates are not immediately available for reference resolution, they have to be actively searched for in the visual environment. However, the time available for this purpose is limited by the ongoing comprehension process that is driven by the linguistic input. In principle, the search could terminate after the target object has been uniquely identified, but at this point in time, it is usually not clear whether the object found is the only fitting one (Hollingworth, 2012), and completely or partially occluded target objects have also to be taken into account. Psychophysical experiments indicate that humans implicitly utilize a threshold to stop visual search (Wolfe, 2012). This threshold is set based on the gist of the scene, which factors in the number of the targets searched for (if it is known), the properties of the target(s), the distinctiveness of the properties, as well as the complexity of the environment and the task at hand. Memory capacity, which is required to keep track of the items, is another factor, especially when there are many more instances that match the search criteria fully or partially. Revisiting the visual objects repeatedly is inhibited and humans are able to adapt the threshold to changing conditions very flexibly.

## 6. Crossmodal Interaction of Language and Vision

A growing body of psycholinguistic evidence gives rise to the assumption that crossmodal integration is more than just a simple procedure of information selection or merging. Instead, it requires intense interactions between independent but closely cooperating processing components. But how, when and (in more recent research also) to what extent this crossmodal interaction occurs is still under investigation.

Crossmodal integration can occur at different levels, from multi-sensory fusion (e.g., audio-visual, audio-tactile or visio-tactile) to higher-level comprehension processes like language understanding. Usually processing is biased with one modality dominating the other one, e.g., the evolutionary acquired dominance of the visual modality over the auditory one for sound-source localization. However, this dominance can be neutralized or even reversed when the dominant modality is not reliable (Witten and Knudsen, 2005; King, 2009).

While perceptual phenomena have a noticeable influence, the integration of linguistic and visual information mostly happens on the conceptual level. Syntax-first approaches assume a privileged role of grammar that is applied in a modular fashion without external influence from other information sources. In case of a crossmodal conflict, the syntax-first approach assumes that only a structure which is licensed by the grammar is chosen (e.g., Frazier and Clifton, 2001, 2006). As a consequence, these theories predict a strict temporal order of processing steps with syntactic constraints being applied first and others later.

Constraint-based approaches (also known as interactive models) suggest a different view, namely that syntactic structures are activated in parallel, taking into account all the relevant information from the available modalities at the same time (e.g., Tanenhaus et al., 1995). From this point of view, all the available contributions can be understood as (contextual) constraints on the language comprehension process, and they exert their influence on the eventual outcome, a consolidated meaning representation, which evolves over time as the utterance unfolds. In constraint-based approaches, the role of extra-linguistic evidence, for example visual percepts, prior experiences or prototypical knowledge about the world, in principle does not differ from the genuine linguistic influence. Crossmodal language comprehension is considered a richly interactive cognitive process of constraint satisfaction that mediates between the different, possibly even conflicting requirements (Louwerse, 2008; Ferreira et al., 2013; Spivey and Huette, 2013). This high degree of openness seems to contribute much to the flexibility, economy and robustness of human sentence comprehension.

Data from several studies has revealed that the assumption that all the constraining information is available at once was too simplistic. Coco and Keller (2015) investigated which kinds of information affect different comprehension processes. In a set of three experiments, they manipulated only the visual saliency, only the linguistic saliency (by means of prosodic markers) and both of them together. The results revealed (1) that visual saliency narrows down the visual search space toward a target, but does not have a direct role on linguistic ambiguity resolution, (2) that intonational breaks add prominence to linguistic referents and favor one interpretation over the other, and (3) that no statistical effect between the two modalities has been found, although they complement each other and both contribute to the overall understanding of the sentence by playing a role in different aspects of language processing.

A more detailed view on the interplay of world knowledge, visual information and linguistic expressions was presented by Knoeferle and Crocker (2006). They showed that people indeed use world knowledge, for example, to assign thematic roles. In case this assignment contradicts the visual context, the information from the visual world will outweigh the lexical biases induced by world knowledge. Similarly, Mirković and Altmann (2019) showed that the visual information is used immediately, but constraints based on inferences from world knowledge come into play later on. Another recent study, targeting the task of meaning recovery from acoustically noisy speech comprehension in German, demonstrated how these two sources (general world knowledge and situation-specific cues) interact with each other while forming the interpretation (Alaçam, 2019). Typical object features and affordances (for example, tables have the affordance of putting things on top of it) can be learned by exposure to daily objects. Episodic affordances [such as being available (empty/occupied)], on the other hand, are closely tied to the situation at hand most of the time. The results of the study show that, in case of conflicting cues, the episodic affordances informed by the current situational information will kick in and influence the interpretation toward the less-expected one. The results also indicate that if there is a strong bias toward the default case (based on real-world contingencies), then situation-specific information may not always be strong enough to override it. From the design perspective of crossmodal natural language processing, this finding informs us that a situated language processing system should not only incorporate the prototypical affordances on the world-knowledge level, but also be able to filter them based on their situation-specific features to achieve a correct evaluation of the described situation.

Considering the benefits of crossmodal interaction in human situated language processing, such as mutual support or guidance, combining the data streams in an interactive manner instead of fusing them seems to be a promising approach. Actually, there is a broad range of possibilities for language and vision to interact, which have already been or could be tried. They can be classified along a number of different dimensions:

- Crossmodal integration can range from maintaining independent, but interacting representations for each modality to having a single, common representation. The latter is not interactive since any independence gets lost. We discuss such approaches here because they are an often used computational approach and reveal initial findings, for instance integrating two modalities improves system performance compared to using only one.
- Either crossmodal interaction takes place just once, or both modalities modulate each other repeatedly.
- The interaction can occur at any point, ranging from an early stage to a later one.
- Either only one modality influences the other or both modalities influence one another mutually.
- If the influence is mutual, it can be realized by means of a bidirectional mapping or with two separate mechanisms, one for each direction of influence.

Most systems discussed throughout this paper do not address all of these distinctions, and they sometimes apply simplifying assumptions, such as using manually annotated images as visual input. Also, not all approaches are designed solely for the general purpose of language understanding. Often, they deal with specific tasks that, among other things, require at least rudimentary language comprehension capabilities, for example sentiment analysis or question answering. It can be assumed that the use visual information in these systems, to a certain degree, compensates for the lack of genuine language understanding capabilities.

Yu and Jiang (2019) reported that using both modalities together is more effective than using them individually with respect to the task of Target-Oriented Sentiment Classification, which determines the sentiment over different individuals, for instance people or places. For this task, the authors propose a neural network that combines the BERT architecture (Devlin et al., 2019) with a target attention mechanism and self-attention layers to model intra- and inter-modality alignments. Table 2 shows that combining linguistic and visual information results in an improved system performance compared to using these modalities in isolation. This holds true for the two publicly available data sets Twitter-15 (Zhang et al., 2018) and Twitter-17 (Lu et al., 2018).

**Table 2:** Comparison of using linguistic and visual information individually or together for Target-Oriented Sentiment Classification, evaluated by Yu and Jiang (2019) on two publicly available data sets.

	**Accuracy**	
**Modality**	**Twitter-15**	**Twitter-17**
Language	74.3%	68.9%
Vision	59.9%	58.6%
Language + Vision^a^	**77.2%**	**70.5%**

a *Different configurations of the neural network architecture were used for each test data set*.

While Yu and Jiang (2019) did not consider any acoustic input, in particular no prosodic information, the results will be contradictory if prosody is included as well. The benefit of prosodic information strongly depends on the task to be achieved. Table 3 compares two crossmodal approaches for sentiment detection that were both evaluated on the CMU-MOSI data set (Zadeh et al., 2016). In addition to the linguistic and visual input, this data set contains prosodic features as a third input modality. Zadeh et al. (2017) showed that combining all available modalities improved the overall system performance, compared to using any of the input modalities alone. The authors applied a Tensor Fusion Network, which is a neural network approach that results in a common, crossmodal representation based on a combination of features for unimodal, bimodal and trimodal interactions. The accuracy of the crossmodal classification improves by 2.3 percentage points compared to the purely linguistic model, by 10.3 percentage points compared to the visual one and by 12.0 percentage points compared to the acoustic one. Poria et al. (2017), who utilized an LSTM-based approach, reported that these improvements mainly result from combining linguistic and visual input. In contrast, adding prosodic features only yields an accuracy improvement of 0.1 percentage points, which suggests that prosody does not significantly contribute to the particular case of sentiment analysis.

**Table 3:** Comparison of three crossmodal sentiment analysis approaches evaluated on the CMU-MOSI data set, one being incremental (Liang et al., 2018) as opposed to two non-incremental ones.

	**Accuracy**		
**Modality**	**Zadeh et al. (2017)**	**Poria et al. (2017)**	**Liang et al. (2018)**
Language	74.8%	78.1%	–
Vision	66.8%	55.8%	–
Prosody	65.1%	60.3%	–
Language + Vision	–	**80.2%**	–
Language + Prosody	–	79.3%	–
Vision + Prosody	–	62.2%	–
Language + Vision + Prosody	**77.1%**	**80.3%**	78.4%

Whereas, sentiment analysis does not benefit from using prosody, Poria et al. (2017) found a strong influence when applying their system to the task of emotion recognition. Table 4 contains their recognition results for four typical emotions that were obtained on the IEMOCAP data set (Busso et al., 2008). Again, they achieved better results using crossmodal features. The accuracy increases by up to 2.5 percentage points compared to using the linguistic features alone, by 28.6 percentage points compared to using only the visual ones and by 27.6 percentage points compared to employing only the acoustic ones. In contrast to their sentiment analysis study, including prosody leads to better results. The accuracy improves from 0.8 (*Angry*) up to 1.7 percentage points (*Neutral*) compared to only combining language and vision. For *Sad*, either visual or prosodic information is required whereas the combination of all modalities does not further improve the accuracy. For *Happy* though, language alone is sufficient. Considering the contradictory findings for sentiment analysis and emotion recognition, we have to assume that prosody is able to improve system performance only for particular tasks. Since many recent language and vision data sets do not contain any prosodic annotations, this hypothesis is hard to verify at the moment.

**Table 4:** Comparison of two crossmodal emotion recognition approaches evaluated for four emotions of the IEMOCAP data set, one being incremental (Liang et al., 2018) as opposed to a non-incremental one (Poria et al., 2017).

		**Accuracy**			
**Approach**	**Modality**	**Angry**	**Happy**	**Sad**	**Neutral**
	Language	76.1%	**79.0%**	76.2%	67.4%
	Vision	53.2%	58.2%	55.5%	51.3%
	Prosody	58.4%	60.5%	61.4%	52.3%
Poria et al. (2017)	Language + Vision	77.2%	**79.0%**	**78.4%**	68.2%
	Language + Prosody	77.2%	**79.1%**	**78.1%**	69.1%
	Vision + Prosody	68.2%	72.0%	70.4%	62.4%
	Language + Vision + Prosody	**78.0%**	**79.3%**	**78.3%**	**69.9%**
Liang et al. (2018)	Language + Vision + Prosody	**85.1%**	**87.5%**	**83.8%**	69.5%

Not only the choice of input features but also how the modalities are integrated affects system performance. Tan and Bansal (2018) used crossmodal interaction by means of mutual attention mechanisms so that language and vision can exert their influence on one another. For the visual reasoning task of the NLVR data set (Suhr et al., 2017), the accuracy improved to 69.7 % for using mutual attention compared to 58.7 % for applying the neural network architecture without any interaction between the two modalities. Comparable results were achieved by Yao et al. (2018), who proposed a similar approach where the interaction occurs repeatedly. Although more evidence is required, these results suggest that separate representations for language and vision, which interact with and influence each other early on, are advantageous compared to the late fusion of unimodal representations without any prior interaction.

While the aforementioned approaches in this section do not consider the possibility that one of the modalities could sometimes not be available or accessible, McCrae (2009) and Crocker et al. (2010) studied this issue by enabling their systems to produce results based on the linguistic input alone. Also, Kiros et al. (2018) proposed a system architecture that can fall back to processing only the linguistic input in case of missing visual information. The authors perform a top-*k* image web search for each word, extract neural features from each image and combine them with pretrained word embeddings via a gating function that controls the influence of the two modalities. If, for example, the web search failed, the system will fall back on the word embeddings alone. Alternatively, Wang et al. (2018), who investigated how to deal with information that is either incomplete or missing entirely in one modality, suggested training a crossmodal system to account for such cases. They reconstructed the missing data based on intra- and inter-modal correlations, which are learned during training by means of a modality dropout simulating the information deficit in one channel.

For fundamental reasons, a learning system can only acquire the kind of desired synergy between linguistic and visual contributions if they are well represented in the training data. Unfortunately, a study by Shekhar et al. (2017) has shown that many existing multi-modal data sets for visual question answering (VQA), a particular version of visual reasoning, possess a certain linguistic bias. The authors found that some questions can already be answered correctly without taking the image into account at all. As a countermeasure, they designed a new unbiased data set that can only be processed successfully if language and vision are modeled in an unbiased manner. Since different state-of-the-art VQA systems performed poorly on this data set, the authors concluded that this indicates a lack of proper integration of language and vision in these approaches. Still, further investigations are required to find out what makes multi-modal data sets well-suited for the study of crossmodal interaction and how they can be compiled to not suffer from any implicit prior preference.

Although there are many different ways to combine language and vision with respect to the aforementioned classification criteria, no optimal solution that fits all the requirements does exist. A consistent finding across the different experiments has been that combining linguistic and visual information improves system performance and that this effect increases when both input channels mutually influence each other compared to them being integrated without interacting.

## 7. Incrementality

Human language processing occurs over time irrespective of its modes, be it written or spoken, or be it comprehended or produced. Incremental processing is one of the key factors that makes natural language conversation fluent and robust at the same time. It becomes a necessity because the length of the utterance to be dealt with is not known in advance and the state of affairs may change while the utterance unfolds. Being able to make sense of the initial parts of an utterance as early as possible allows the listener to respond to the incoming utterance in a timely manner, either by replying with an appropriate verbal response, such as producing a back-channel signal (like nodding, raising one's eye-brows, or looking at a possible object of reference), or by preparing and initiating an appropriate problem-related action; see Crocker (1999) for a review.

Incremental processing is particularly valuable for early reference resolution. Reliable hypotheses about suitable candidates in the visual environment reduce the space of possible alternative interpretations, which may save computational effort. Moreover, a closer inspection of the candidate and its visual surrounding may contribute additional information that supports the ongoing comprehension process, and therefore has the potential to create the feedback loop for mutual benefit between the linguistic and the visual channel. If strong enough evidence is contributed by another modality, it will even lead to early decisions on sentence structures and referential relationships without realizing that another (linguistic) interpretation would be possible (Tanenhaus et al., 1995; Christianson et al., 2001; Knoeferle et al., 2005; Altmann and Mirković, 2009).

Incrementality can be observed on all levels of linguistic granularity. Words are identified at a very early point in time as soon as the available phonetic and contextual information is sufficient to make a certain enough choice, usually during or shortly after the very first phoneme of the word (Marslen-Wilson and Welsh, 1978). Less obvious is the incremental nature of phonetic perception on the suprasegmental level. But even there, prosodic signals provide additional information to resolve ambiguities and predict the upcoming structure (Bailey and Ferreira, 2007; Snedeker and Yuan, 2008; Coco and Keller, 2015). These cues also allow the listener to estimate the distance to the next point in time when she can interrupt the speaker without risking to be unpolite. Moreover, changing the speech rate between the determiner and the noun during an indefinite noun phrase was shown to have an effect on perceiving the determiner (or ignoring it at all) and on understanding the noun phrase (Brown et al., 2012). Furthermore, contrastive intonation contours seem to be processed incrementally, and their processing is guided by the contextual cues during spoken language understanding (Weber et al., 2006; Kurumada et al., 2014). Not just phonetic cues but also visual information like facial expressions seems to have an immediate impact on sentence processing, facilitating early reference resolution in an incremental manner (Carminati and Knoeferle, 2013; Graham et al., 2017).

From a technical point of view, incremental (online) processing can be distinguished from batch mode (offline) processing. While a system in batch mode waits for the whole input being available before attempting to analyze it, an incremental analysis integrates partial input into a (coherent) processing result as soon as it becomes available. For an outside observer, batch mode processing is equivalent to ignoring the dynamic nature of the input data completely, and therefore this type of processing is not able to explain the dynamics of human language comprehension. We only mention it here to highlight the contrast between human behavior and traditional computational solutions. Incremental processing, on the other hand, reflects the dynamic characteristics of the input in its output: The results produced evolve over time like the input does. A language comprehension system, for instance, processes the input word-by-word constructing semantic relations (possibly even only partially instantiated ones) between linguistic and visual entities as soon as possible.

The dynamics of incremental decision taking ranges from greedy (monotonic) approaches that extend previous partial analyses without ever changing them to pseudo-incremental ones, ignoring all previous results and always analyzing the entire input up to the current increment anew. Truly incremental ones take previous solutions into account and revise them according to the new information just becoming available.

Forcing the language comprehension process to take its decisions as early as possible comes at the price of making intermediate interpretations less certain and more ambiguous because large portions of the linguistic input are not yet available at the point in time when the decision has to be taken. In effect, responsiveness is traded against reliability and in case of wrong decisions, a reanalysis has to be initiated. In the long run, the additional information that is made available from the visual channel by the closely time-locked interaction between the two input channels might overcompensate this effect. Evidence from the human model as well as from computational systems shows that committing to an initial interpretation early on and revising it whenever this becomes necessary is often the more successful strategy compared to a wait-and-see approach (Ferreira, 2003; Baumann et al., 2013).

For English, the phenomenon of temporal ambiguities caused by incremental processing has been investigated most often by means of reduced relative clause constructions. The two sentences “*The pills brought no relief.”* and “*The pills brought by the nurse helped a lot.”* share a common initial part; thus, they initially look the same but later diverge into two constructions that are preferred to different degrees. Despite this superficial similarity, the verb *brought* is actually part of two different kinds of verb groups, either in the active voice (*brought no relief*) or the passive voice (*brought by the nurse*), where the second one is part of a (reduced) relative clause. As a consequence, *the pills* are either the subject, i.e., in that particular case the Agent of the verb *to bring*, or its direct object, i.e., the Theme. Since humans prefer the first reading, they have to revise their interpretation in the second case.

An early artificial approach that deals with partial input was proposed by Brick and Scheutz (2007). Their system, which they claimed to be psychologically plausible, is able to perform actions, like grasping, and provides feedback at an early point in time. Additionally, it can deal with ambiguous utterances as well as references. The system is built upon an incremental semantic module based on constraint propagation that integrates linguistic knowledge with perceptual information from the visual channel. After each increment, reference resolution is performed. If a unique referent is found, the system will start to react. In case of an ambiguity, it will continue with the most plausible interpretation until the unfolding information requires a revision.

One problem of processing sentences incrementally is caused by possible dependencies between incrementality and crossmodality, which have an impact on the system performance that is hard to assess. Liang et al. (2018) proposed a neural architecture (Recurrent Multistage Fusion Network) for incremental processing that, first, processes each modality individually to capture intra-modal dependencies at each time point. Then, all modalities are fused into a common, crossmodal representation. Finally, this inter-modal representation of time point *t* is fed back into the intra-modal representations of step *t* + 1 before the procedure is called again for that next step. The architecture is incremental since it processes the linguistic input word-by-word, with the other input streams segmented accordingly. It was applied to different tasks including sentiment analysis and emotion recognition. Compared to the non-incremental approach of Poria et al. (2017), the accuracy increased for all emotions by at least 5.5 percentage points (see Table 4), except the emotion class *Neutral*, which decreased by 0.4 percentage points. For sentiment analysis, the accuracy decreased by 1.9 percentage points compared to (Poria et al., 2017) (see Table 3). Although incrementality enables a system to react at an early point in time, there is a potential trade-off with respect to the overall system performance that one should be aware of when developing artificial solutions that are both crossmodal and incremental. The most influential factors that could explain these contradictory results still need to be determined.

Standard evaluation metrics for natural language processing systems do not take the dynamic nature of incremental results into account. They only evaluate the quality at a fixed point in time (usually after completion of the computation) and they are not able to describe the timeliness of a system. Therefore, Schlangen et al. (2009) proposed a number of novel measures to evaluate the intermediate results of incremental processing with respect to when a systems takes decisions and how often it changes them. Timeliness, for instance, refers to the delay of the system output, and non-monotonicity measures the portion of the intermediate results that will be part of the final result. Unfortunately, these metrics do not quantify the quality of the intermediate results. To overcome this deficiency, Beuck et al. (2013) suggested to use temporal quality profiles, which can be determined by a point-wise analysis of intermediate results in terms of accuracy. These profiles describe the reliability of attaching the *n* most recent words in a window left of the current point in time and usually show a fairly low reliability for the newest word, which increases the older the hypotheses are. A detailed description of these performance characteristics and a discussion of the inherent trade-offs of an incremental system can be found in Köhn (2018).

## 8. Prediction

While incremental comprehension aims at integrating all the already available pieces of input into a coherent tentative result, predictive processing takes a more radical approach by trying to produce output based on its expectations about the upcoming observations. Predictions can be checked against the visual evidence proactively and they can guide the visual attention toward the relevant entities in the environment, even though the referring expression is still missing or underspecified at that point in time. Thus, predictive processing amplifies the advantages of incrementality even further. It also minimizes the temporal delay between perceiving the initial part of an utterance and understanding it, effectively providing an additional gain in responsiveness at the price of taking more risky decisions and the need to possibly correct them later on. The quality of predictions can be calculated by precision and recall (Beuck et al., 2013).

Predictions are guided by an incomplete or unconnected structural interpretation of the already processed linguistic input. They may, for instance, concern the most plausible filler of a thematic role or they can help to create a fully connected, thereby more expressive meaning representation when a concept connecting two other ones is still missing. Having predictions available not only speeds up reference resolution, but also helps the listener to disambiguate the role assignment itself.

Expectations about the most likely thematic role fillers usually are introduced by a verb or a comparable lexical item (e.g., Trueswell et al., 1993; Altmann and Kamide, 1999; Chambers et al., 2004). These expectations can be used, for example, to determine the Theme of a particular action. Altmann and Kamide (1999) have found that listeners are able to predict the complements of a verb based on its selectional constraints and the affordances of the possible role fillers. When people hear the verb *break*, for instance, their attention is directed toward breakable objects in the scene. Similar to verbs, some non-verbs also generate expectations about what may follow (McRae et al., 2001).

A natural language understanding system whose comprehension and prediction capability improved by including predictions derived from affordances into the decision taking process was presented by Gorniak and Roy (2007). In their study, they employ a probabilistic, hierarchical approach for plan recognition and, thereby, integrate language, situation-specific knowledge from the visual channel and general world knowledge, namely action affordances. Their method requires predictive parsing, which they realize by means of a Combinatory Categorial Grammar in combination with affordance filters. After processing a noun, a subset of actions remains that can be applied to all past, present and future states. For instance, *gate* relates to opening, locking or walking through. In contrast, a verb restricts the set of possible objects: For example, *open* refers to objects that can be opened.

The other kind of prediction is used to create a connected structural description for the partial linguistic input. This becomes necessary in a situation where the attribute of an object is already available, but not yet the noun to which it refers: “*I'll take the white…”*. Here, the prediction helps to establish the semantic connection between the verb *to take* and the color attribute *white* by means of the still unknown, but already predictable object role.

Predictions can also be triggered by salient characteristics of the visual environment: The grouping of objects may help to anticipate possible completions of a coordinated structure (*plates and…*), or the display of a dominating action may help to predict the predicate together with all the restrictions for its arguments. If the environment is dynamic, one can also predict possible updates, like the probable outcome of an action that changes the state of an entity.

Knoeferle et al. (2005) demonstrated the predictive nature of sentence comprehension using German sentences with Subject-Object ambiguities that directly map to an ambiguous Agent/Patient assignment. Each visual scene to which a sentence refers depicts two actions and three characters. Two different sentence patterns have been compared either in an unmarked word order (Subject-Verb-Object) or in a marked word order (Object-Verb-Subject). Due to the case ambiguity of German feminine nouns (nominative or accusative), it is not possible to decide whether the first noun phrase in the sentence is an Agent or a Patient until the case-marker of the second noun phrase following the verb becomes available. Eye-tracking results showed that in case of the marked word order, the initial role assignment for the first noun phrase creates a conflict with the following assignment for the second one, and a reanalysis becomes necessary. Most interestingly, visual attention already started to move toward the target character before the associated post-verbal noun phrase actually appeared, i.e., while the verb was still being spoken. This clearly signals that reference resolution for the second noun phrase was not based on the observation of the phrase itself but on its prediction induced by the verb. A follow-up study on the same data set but using event-related brain potentials (ERPs) as a research methodology also confirmed this conclusion (Knoeferle et al., 2007).

Based on the aforementioned studies, Knoeferle and Crocker (2007) developed the Coordinated Interplay Account, a recurrent connectionist network, which models language-mediated visual attention and sentence interpretation. With a direct correspondence to the psycholinguistic findings, the model can successfully predict human behavior and neuro-imaging results described previously (Knoeferle et al., 2005; Knoeferle and Crocker, 2007). In particular, it can correctly resolve ambiguous thematic role assignments at the same point in time as people do (Crocker et al., 2010). The network assigns thematic roles incrementally in the unimodal as well as in the crossmodal one. It consists of three subparts: (1) unimodal sentence comprehension, (2) modulation of visual attention by the partial linguistic analyses and (3) the modulation of the sentence interpretation using the additional information gathered from the visual scene. All three subparts maintain independent representations, and crossmodal reference resolution is realized by means of co-indexation. The approach can deal with scenes that contain more entities than those referred to in the sentence. The model is also robust in the absence of visual input (Mayberry et al., 2009), since it can capture stereotypical associations between agents and their actions if they appear frequently enough in the training data. Integrating the visual information will improve the interpretation in case these associations are difficult to extract or irrelevant. It should be noted, though, that the system utilizes a model structure that is specifically tailored to predict the experimental data. Therefore, the system does not possess any kind of general language comprehension capability.

The objects or events that are not directly referred to in an utterance will attract an increased amount of attention if they are inferred either from what has been said so far or from the concurrent visual information (Dahan and Tanenhaus, 2005). Altmann and Kamide (2007) investigated this relationship by manipulating the tense of the main verb. The visual stimuli contained a cat (i. e. an animal that could kill), some mice (i.e., animals that can be killed by a cat, but are still alive), a pile of feathers (i.e., the remainder of a bird that has been killed) and some distractor objects. Two different versions of a spoken sentence were presented, either “*The cat will kill all of the mice.”* or “*The cat has killedall of the birds.”* While more fixations on the mice, after the onset of the auxiliary verb, have been observed in the former condition, more fixations on the feathers occurred in the latter case, although the feathers cannot be the target of a killing action, but still overlap with the conceptual requirements of the verb. Thus, the participants still anticipated *feathers* as the Theme using contextual information which the authors call real-world contingencies. In such cases, the mapping of language onto the scene becomes even more challenging but crucial for natural language processing solutions since the temporal structure of the events entailed by the sentence or change of state (namely objects that disappear or change their appearance such as things which have been eaten up or will be constructed) needs to be considered.

Even the objects that have no semantical relation to the spoken words are able to attract attention to themselves. To study to which degree the aforementioned findings (Knoeferle et al., 2005) are influenced by the visual complexity of the visual scene, Alaçam et al. (2019) followed the same experimental paradigm and used the same sentence patterns (unmarked and marked word orders). The visual stimuli differed from those used in Knoeferle et al. (2005) by a meaningfully structured background containing a substantial amount of distractors (c.f. Figure 1), additional background objects and an additional character acting on the ambiguous Agent/Patient character. The results replicate the findings previously reported in Knoeferle et al. (2005) that participants are garden-pathed when they hear a sentence in Object-Verb-Subject order. Although none of the visual manipulations is directly related to the entities mentioned in the sentence, the amount of fixations on the target is still influenced by the visual clutter regarding the irrelevant entities in the scene. The overall fixation rate decreases when the complexity increases with a stronger effect caused by the additional character compared to additional background objects.

## 9. Heuristic Decision Taking

The studies on structural prediction and ambiguity resolution discussed above have been carried out within relatively simple visual and linguistic settings, where the relationships between events and entities can be extracted easily. More recently, the effect of visual complexity has increasingly attracted attention. As the complexity (either of the visual context or the task) grows, using the visual information to narrow down the space of possible linguistic interpretations becomes more difficult. In such cases, subjects either tend to choose a more passive strategy, such as waiting for more detailed information about the entities mentioned in the utterance instead of taking decisions based on risky anticipations (Ferreira et al., 2013). Alternatively, they can resort to simple heuristics, like choosing an interpretation which is in line with stereotypical semantic information or the visual world, even though this interpretation requires to accept grammatically unacceptable syntactic structures (MacWhinney et al., 1984; Christianson et al., 2001). In a Dutch noun phrase, the prenominal adjective(s) as well as the head noun are gender-marked, and the gender of the adjective(s) has to agree with the gender of the noun. Normally, the gender information of the preceding adjective is used to predict the target object before the corresponding noun has been uttered (Van Berkum et al., 2005). Instead, Brysbaert and Mitchell (2000) found that people sometimes are insensitive to this kind of morphological cue. In their study, subjects have chosen good enough representations with a better semantic fit, but ignored the disambiguating gender information that contradicts their interpretation.

Such kind of heuristic decision taking plays a crucial role in human cognition as comprehensively discussed in Gigerenzer (2000) and Gigerenzer (2008). In complex enough tasks, reaching a decision by considering all possible options is unrealistic due to temporal or memory-related resource limitations. Thus, there must be a cognitive mechanism that is able to abandon processing and to settle the issue as soon as a sufficiently high degree of confidence has been reached.

Ferreira (2003) argues that spoken sentence comprehension is an inherently demanding task that involves complex sequential decision taking and is affected by both uncertainty about the current input and a lack of knowledge about the upcoming material. Thus, enforcing consistency among the sequential decisions is not always feasible and people resort to fast and frugal heuristics, thereby producing good enough representations. The assumption of good enough representations also provides a valid explanation for the (partial) success of conversations in noisy environments. In such a scenario, instead of waiting for or requesting intelligible spoken input, combining the uncertain information from the linguistic and the visual channel would be a more effective comprehension strategy.

A computational approach discussed above that applies heuristics can be found in Baumgärtner (2013). Its constraints rely on attachment heuristics. Some attachments are preferred compared to others that are nonetheless plausible. For instance, low attachments occur more often in German than higher ones. Hence, the latter incur a small penalty. In case no alternative is possible, these mild constraint violations are accepted, though, and deemed as a good enough solution. Also, the constraint solving mechanism will stop after a predefined number of steps or if only minimal improvements are made. In such cases, the solution will also be viewed as good enough.

The Late Assignment of Syntax hypothesis (Townsend and Bever, 2001) addresses the role of heuristics on language comprehension from a theoretical perspective. According to this theory, sentence processing is performed in two steps. First, a pseudo-parser tries to obtain a very shallow interpretation based on syntactic frequencies and semantic associations, for example, a heuristic captures the tendency to treat the first argument in a sentence as Agent, and the second argument as Patient. In the second step, a full-fledged and therefore more time-consuming parser is applied which is guided by the results of the shallow one. In case of resource limitations, the results of the shallow parser are taken, which could of course be wrong. Occasionally, the results of the two different parsers might not agree. Then the system needs to reconcile them and decide on the final, possibly still erroneous interpretation.

## 10. Conclusions

It is commonplace that language comprehension takes advantage of the availability of crossmodal information. Indeed, recent psycholinguistic research as well as the development of computational language comprehension systems have confirmed this assumption and contributed a number of valuable insights into how this added benefit comes about and what its prerequisites are:

- Language comprehension benefits from being sensitive to extra-linguistic information about the kind of entities in the surrounding world, their spatial relationships, the events they take part in, and the general or episodic affordances they offer. Because it is situation-specific, this information provides a welcome complement to the more static type of knowledge that can be extracted from large-scale linguistic data collections. It helps, for instance, to resolve ambiguous thematic role assignments, to correctly attach words and phrases, and eventually to determine the most likely intention of the speaker.
- The interplay between linguistic and visual processing components seems to be based on interaction rather than fusion. Interaction preserves the autonomy of the modalities while providing the possibility for information exchange and reconciliation from the very first moment. Avoiding a separate *post-hoc* component for information selection and combination, such an architecture improves the robustness against information deficits in one of the channels. This property might have contributed to the gains in output quality found in interactive computational solutions.
- Language processing can profit most from the available visual information if it proceeds in an incremental and predictive manner. Incrementality and prediction not only make a comprehension system more responsive, but can also guide the visual attention almost instantaneously to the relevant areas in the visual environment, facilitating early reference resolution and a rapid extraction of additional disambiguating cues from the visual channel. The sources that inform prediction are the same as the ones that are used for language comprehension in general. They range from purely language-internal ones, such as the lexically induced valency requirements of verbs, to the purely visual, which can also be used for ambiguity resolution.

Building visually informed language comprehension systems also requires a major effort to collect and annotate appropriate data. On the one hand, this data should be free of bias, since only then a training procedure will be able to extract the relevant associations between the modalities involved. On the other hand, it has to conform to the requirements of incremental and predictive processing, which in general is non-monotonic and often requires modifying already produced, tentative output in the light of additional input becoming available later. Ignoring these temporal aspects might mislead model training toward wrong crossmodal mappings. Thus, novel annotation schemes, suitable data transformation approaches and sophisticated training procedures will be needed.

While still falling short of what the human model can accomplish, artificial systems have made significant advances in many of the above-mentioned respects. Models and algorithms for incremental and predictive processing have been developed, and at least in restricted application scenarios a beneficial impact of crossmodal interaction has been found. Nevertheless, all the approaches we reviewed focus on selected aspects of crossmodal language comprehension, i.e.,

- addressing conceptual integration and structural disambiguation, but ignoring the inherent perceptual uncertainty of speech and vision,
- experimenting with crossmodal interaction without considering the dynamic nature of incremental processing, or
- aiming at shallow processing tasks, like sentiment detection or emotion recognition, avoiding any problems with reference resolution, meaning analysis and intention detection.

However, such an isolated consideration ignores the many dependencies which exist between the different aspects and which might have a significant impact on the overall performance of a system. The full potential of crossmodal language comprehension will only become available if these aspects are dealt with in an integrated manner. Visually guided sentence parsing, visually or lexically induced prediction of upcoming linguistic structures, continuous interaction between the modalities, linguistically guided visual attention, etc. all contribute in different but complementary ways to the ongoing process of language comprehension. None of them alone will be sufficient to achieve human-like natural language communication behavior. While the first successful attempts have been made to implement human-inspired processing mechanisms in artificial agents, their interplay is not well understood, neither in the human brain nor *in silico*. Only their combination in a single, well-balanced architecture where the modalities can interact with each other on small enough input increments will pave the way toward behavior that comes close to the language processing capabilities of the human model.

## Author Contributions

All four authors have been equally much involved in the discussion of the structure, the general content, and the final integration. In particular: ÖA, XL, WM, and TS: introduction, visual guidance, search, and conclusions. ÖA, WM, and TS: speaker intention, resolution of linguistic ambiguities, crossmodal reference resolution, crossmodal interaction of language and vision, incrementality, prediction, and heuristic decision taking.

### Conflict of Interest

The authors declare that the research was conducted in the absence of any commercial or financial relationships that could be construed as a potential conflict of interest.

## References

[B1] Alaçam O. (2019). Enhancing natural language understanding through cross-modal interaction: meaning recovery from acoustically noisy speech, in Proceedings of the 22nd Nordic Conference on Computational Linguistics, eds Hartmann M. Barbara P. (Turku: Linköping University Electronic Press), 272–280.

[B2] Alaçam O. Menzel W. Staron T. (2019). How does visual complexity influence predictive language processing in a situated context?, in Preliminary Proceedings of the 15th Conference on Natural Language Processing (KONVENS 2019) (Erlangen: German Society for Computational Linguistics & Language Technology), 256–261.

[B3] Altmann G. T. M. (2017). Abstraction and generalization in statistical learning: implications for the relationship between semantic types and episodic tokens. Philos. Trans. R. Soc. B Biol. Sci. 372:20160060. 10.1098/rstb.2016.0060 27872378 PMC5124085

[B4] Altmann G. T. M. Kamide Y. (1999). Incremental interpretation at verbs: restricting the domain of subsequent reference. Cognition 73, 247–264. 10.1016/S0010-0277(99)00059-1 10585516

[B5] Altmann G. T. M. Kamide Y. (2007). The real-time mediation of visual attention by language and world knowledge: linking anticipatory (and other) eye movements to linguistic processing. J. Mem. Lang. 57, 502–518. 10.1016/j.jml.2006.12.004

[B6] Altmann G. T. M. Mirković J. (2009). Incrementality and prediction in human sentence processing. Cogn. Sci. 33, 583–609. 10.1111/j.1551-6709.2009.01022.x 20396405 PMC2854821

[B7] Atkinson E. Wagers M. W. Lidz J. Phillips C. Omaki A. (2018). Developing incrementality in filler-gap dependency processing. Cognition 179, 132–149. 10.1016/j.cognition.2018.05.022 29936344

[B8] Bailey K. G. D. Ferreira F. (2007). Chapter 22: The processing of filled pause disfluencies in the visual world, in Eye Movements, eds Van Gompel R. P. G. Fischer M. H. Murray W. S. Hill R. L. (Oxford: Elsevier), 487–502.

[B9] Baumann T. Paetzel M. Schlesinger P. Menzel W. (2013). Using affordances to shape the interaction in a hybrid spoken dialog system, in Studientexte zur Sprachkommunikation: Elektronische Sprachsignalverarbeitung 2013, ed Wagner P. (Dresden: TUD Press), 12–19.

[B10] Baumgärtner C. (2013). On-line cross-modal context integration for natural language parsing (Ph.D. Thesis). Dept. of Computer Science, University of Hamburg, Hamburg, Germany.

[B11] Beuck N. Köhn A. Menzel W. (2013). Predictive incremental parsing and its evaluation, in Computational Dependency Theory, Vol. 258 of Frontiers in Artificial Intelligence and Applications, eds Gerdes K. Hajičová E. Wanner L. (Amsterdam: IOS Press), 186–206.

[B12] Bloch I. editor (2008). Information Fusion in Signal and Image Processing. Hoboken, NJ: John Wiley & Sons, Inc.

[B13] Brick T. Scheutz M. (2007). Incremental natural language processing for HRI, in 2007 2nd ACM/IEEE International Conference on Human-Robot Interaction (HRI) (Arlington, VA: IEEE), 263–270.

[B14] Brown M. Dilley L. C. Tanenhaus M. K. (2012). Real-time expectations based on context speech rate can cause words to appear or disappear, in Proceedings of the 34th Annual Meeting of the Cognitive Science Society, eds Miyake N. Peebles D. Cooper R. P. (Sapporo: Cognitive Science Society), 1374–1379.

[B15] Brysbaert M. Mitchell D. C. (2000). The failure to use gender information in parsing: a comment on van Berkum, Brown, and Hagoort (1999). J. Psycholinguist. Res. 29, 453–466. 10.1023/A:1005191308387 10723710

[B16] Busso C. Bulut M. Lee C.-C. Kazemzadeh A. Mower E. Kim S. (2008). IEMOCAP: interactive emotional dyadic motion capture database. Lang. Resour. Eval. 42, 335–359. 10.1007/s10579-008-9076-6

[B17] Carminati M. N. Knoeferle P. (2013). Effects of speaker emotional facial expression and listener age on incremental sentence processing. PLoS ONE 8:e72559. 10.1371/journal.pone.0072559 24039781 PMC3765193

[B18] Chambers C. G. Tanenhaus M. K. Magnuson J. S. (2004). Actions and affordances in syntactic ambiguity resolution. J. Exp. Psychol. Learn. Mem. Cogn. 30, 687–696. 10.1037/0278-7393.30.3.687 15099136

[B19] Christianson K. Hollingworth A. Halliwell J. F. Ferreira F. (2001). Thematic roles assigned along the garden path linger. Cogn. Psychol. 42, 368–407. 10.1006/cogp.2001.0752 11368528

[B20] Clark H. H. Schreuder R. Buttrick S. (1983). Common ground and the understanding of demonstrative reference. J. Verbal Learn. Verbal Behav. 22, 245–258. 10.1016/S0022-5371(83)90189-5

[B21] Coco M. I. Keller F. (2015). The interaction of visual and linguistic saliency during syntactic ambiguity resolution. Q. J. Exp. Psychol. 68, 46–74. 10.1080/17470218.2014.936475 25176109

[B22] Crocker M. W. (1999). Chapter 7: Mechanisms for sentence processing, in Language Processing, eds Garrod S. Pickering M. J. (New York, NY; London: Psychology Press), 191–232.

[B23] Crocker M. W. Knoeferle P. Mayberry M. R. (2010). Situated sentence processing: the coordinated interplay account and a neurobehavioral model. Brain Lang. 112, 189–201. 10.1016/j.bandl.2009.03.004 19450874

[B24] Dahan D. Tanenhaus M. K. (2005). Looking at the rope when looking for the snake: conceptually mediated eye movements during spoken-word recognition. Psychon. Bull. Rev. 12, 453–459. 10.3758/BF03193787 16235628

[B25] Devlin J. Chang M.-W. Lee K. Toutanova K. (2019). BERT: pre-training of deep bidirectional transformers for language understanding, in Proceedings of the 2019 Conference of the North American Chapter of the Association for Computational Linguistics: Human Language Technologies, Volume 1 (Long and Short Papers), eds Burstein J. Doran C. Solorio T. (Minneapolis, MN: Association for Computational Linguistics), 4171–4186.

[B26] Duff M. C. Brown-Schmidt S. (2012). The hippocampus and the flexible use and processing of language. Front. Hum. Neurosci. 6:69. 10.3389/fnhum.2012.00069 22493573 PMC3319917

[B27] Engelhardt P. E. Bailey K. G. D. Ferreira F. (2006). Do speakers and listeners observe the Gricean Maxim of Quantity? J. Mem. Lang. 54, 554–573. 10.1016/j.jml.2005.12.009

[B28] Ferreira F. (2003). The misinterpretation of noncanonical sentences. Cogn. Psychol. 47, 164–203. 10.1016/S0010-0285(03)00005-7 12948517

[B29] Ferreira F. Foucart A. Engelhardt P. E. (2013). Language processing in the visual world: effects of preview, visual complexity, and prediction. J. Mem. Lang. 69, 165–182. 10.1016/j.jml.2013.06.001

[B30] Frazier L. Clifton C. Jr. (2001). Parsing coordinates and ellipsis: copy α. Syntax 4, 1–22. 10.1111/1467-9612.00034

[B31] Frazier L. Clifton C. Jr. (2006). Ellipsis and discourse coherence. Linguist. Philos. 29, 315–346. 10.1007/s10988-006-0002-3 16896367 PMC1533872

[B32] Gigerenzer G. (2000). Adaptive Thinking: Rationality in the Real World. Evolution and Cognition. Oxford; New York, NY: Oxford University Press.

[B33] Gigerenzer G. (2008). Why heuristics work. Perspect. Psychol. Sci. 3, 20–29. 10.1111/j.1745-6916.2008.00058.x 26158666

[B34] Gorniak P. Roy D. (2005). Speaking with your sidekick: understanding situated speech in computer role playing games, in Proceedings of the First AAAI Conference on Artificial Intelligence and Interactive Digital Entertainment, AIIDE'05, eds Young R. M. Laird J. (Marina del Rey, CA: AAAI Press), 57–62.

[B35] Gorniak P. Roy D. (2007). Situated language understanding as filtering perceived affordances. Cogn. Sci. 31, 197–231. 10.1080/15326900701221199 21635295

[B36] Graham S. A. San Juan V. Khu M. (2017). Words are not enough: how preschoolers' integration of perspective and emotion informs their referential understanding. J. Child Lang. 44, 500–526. 10.1017/S0305000916000519 27817761

[B37] Gundel J. K. Hedberg N. Zacharski R. (2012). Underspecification of cognitive status in reference production: some empirical predictions. Top. Cogn. Sci. 4, 249–268. 10.1111/j.1756-8765.2012.01184.x 22389129

[B38] Hindy N. C. Altmann G. T. M. Kalenik E. Thompson-Schill S. L. (2012). The effect of object state-changes on event processing: do objects compete with themselves? J. Neurosci. 32, 5795–5803. 10.1523/JNEUROSCI.6294-11.2012 22539841 PMC3368505

[B39] Hindy N. C. Solomon S. H. Altmann G. T. M. Thompson-Schill S. L. (2013). A cortical network for the encoding of object change. Cereb. Cortex 25, 884–894. 10.1093/cercor/bht275 24127425 PMC4366611

[B40] Hollingworth A. (2012). Guidance of visual search by memory and knowledge, in The Influence of Attention, Learning, and Motivation on Visual Search, Nebraska Symposium on, eds MotivationDodd M. D. Flowers J. H. (New York, NY: Springer), 63–89. 10.1007/978-1-4614-4794-8_4 PMC3875155 23437630

[B41] Huettig F. Rommers J. Meyer A. S. (2011). Using the visual world paradigm to study language processing: a review and critical evaluation. Acta Psychol. 137, 151–171. 10.1016/j.actpsy.2010.11.003 21288498

[B42] Itti L. Koch C. (2000). A saliency-based search mechanism for overt and covert shifts of visual attention. Vision Res. 40, 1489–1506. 10.1016/S0042-6989(99)00163-7 10788654

[B43] Kelleher J. D. Costello F. J. (2009). Applying computational models of spatial prepositions to visually situated dialog. Comput. Linguist. 35, 271–306. 10.1162/coli.06-78-prep14

[B44] King A. J. (2009). Visual influences on auditory spatial learning. Philos. Trans. R. Soc. B Biol. Sci. 364, 331–339. 10.1098/rstb.2008.0230 18986967 PMC2674475

[B45] Kiros J. R. Chan W. Hinton G. E. (2018). Illustrative language understanding: Large-scale visual grounding with image search, in Proceedings of the 56th Annual Meeting of the Association for Computational Linguistics (Volume 1: Long Papers), eds Gurevych I. Yusuke M. (Melbourne, QC: Association for Computational Linguistics), 922–933.

[B46] Kitaev N. Klein D. (2017). Where is Misty? Interpreting spatial descriptors by modeling regions in space, in Proceedings of the 2017 Conference on Empirical Methods in Natural Language Processing, eds Palmer M. Hwa R. Riedel S. (Copenhagen: Association for Computational Linguistics), 157–166.

[B47] Knoeferle P. Crocker M. W. (2006). The coordinated interplay of scene, utterance, and world knowledge: evidence from eye tracking. Cogn. Sci. 30, 481–529. 10.1207/s15516709cog0000_65 21702823

[B48] Knoeferle P. Crocker M. W. (2007). The influence of recent scene events on spoken comprehension: evidence from eye movements. J. Mem. Lang. 57, 519–543. 10.1016/j.jml.2007.01.003

[B49] Knoeferle P. Crocker M. W. Scheepers C. Pickering M. J. (2005). The influence of the immediate visual context on incremental thematic role-assignment: evidence from eye-movements in depicted events. Cognition 95, 95–127. 10.1016/j.cognition.2004.03.002 15629475

[B50] Knoeferle P. Habets B. Crocker M. W. Münte T. F. (2007). Visual scenes trigger immediate syntactic reanalysis: evidence from ERPs during situated spoken comprehension. Cereb. Cortex 18, 789–795. 10.1093/cercor/bhm121 17644830

[B51] Köhn A. (2018). Incremental natural language processing: challenges, strategies, and evaluation, in Proceedings of the 27th International Conference on Computational Linguistics, eds Bender E. M. Derczynski L. Isabelle P. (Santa Fe, NM: Association for Computational Linguistics), 2990–3003.

[B52] Koolen R. Goudbeek M. Krahmer E. (2011). Effects of scene variation on referential overspecification, in Proceedings of the 33rd Annual Meeting of the Cognitive Science Society, eds Carlson L. Hoelscher C. Shipley T. F. (Boston, MA: Cognitive Science Society), 1025–1030.

[B53] Kruijff G.-J. M. Lison P. Benjamin T. Jacobsson H. Zender H. Kruijff-Korbayová I. (2010). Chapter 8: Situated dialogue processing for human-robot interaction, in Cognitive Systems, Vol. 8 of *Cognitive Systems Monographs*, eds Christensen H. I. Kruijff G.-J. M. Wyatt J. L. (Berlin; Heidelberg: Springer), 311–364.

[B54] Kurumada C. Brown M. Bibyk S. Pontillo D. F. Tanenhaus M. K. (2014). Is it or isn't it: listeners make rapid use of prosody to infer speaker meanings. Cognition 133, 335–342. 10.1016/j.cognition.2014.05.017 25128792 PMC4163505

[B55] Lazaridou A. Bruni E. Baroni M. (2014). Is this a wampimuk? Cross-modal mapping between distributional semantics and the visual world, in Proceedings of the 52nd Annual Meeting of the Association for Computational Linguistics (Volume 1: Long Papers), eds Toutanova K. Wu H. (Baltimore, MD: Association for Computational Linguistics), 1403–1414.

[B56] Liang P. P. Liu Z. Zadeh A. Morency L.-P. (2018). Multimodal language analysis with recurrent multistage fusion, in Proceedings of the 2018 Conference on Empirical Methods in Natural Language Processing, eds Riloff E. Chiang D. Hockenmaier J. Tsujii J. (Brussels: Association for Computational Linguistics), 150–161.

[B57] Louwerse M. M. (2008). Embodied relations are encoded in language. Psychon. Bull. Rev. 15, 838–844. 10.3758/PBR.15.4.838 18792513

[B58] Lu D. Neves L. Carvalho V. Zhang N. Ji H. (2018). Visual attention model for name tagging in multimodal social media, in Proceedings of the 56th Annual Meeting of the Association for Computational Linguistics (Volume 1: Long Papers), eds Gurevych I. Miyao Y. (Melbourne, QC: Association for Computational Linguistics), 1990–1999.

[B59] MacWhinney B. Bates E. Kliegl R. (1984). Cue validity and sentence interpretation in English, German, and Italian. J. Verbal Learn. Verbal Behav. 23, 127–150. 10.1016/S0022-5371(84)90093-8

[B60] Marslen-Wilson W. D. Welsh A. (1978). Processing interactions and lexical access during word recognition in continuous speech. Cogn. Psychol. 10, 29–63. 10.1016/0010-0285(78)90018-X

[B61] Mayberry M. R. Crocker M. W. Knoeferle P. (2009). Learning to attend: a connectionist model of situated language comprehension. Cogn. Sci. 33, 449–496. 10.1111/j.1551-6709.2009.01019.x 21585477

[B62] McCrae P. (2009). A model for the cross-modal influence of visual context upon language procesing, in Proceedings of the International Conference RANLP-2009, eds Angelova G. Mitkov R. (Borovets: Association for Computational Linguistics), 230–235.

[B63] McCrae P. Menzel W. (2007). Towards a system architecture for integrating cross-modal context in syntactic disambiguation, in Proceedings of the 4th International Workshop on Natural Language Processing and Cognitive Science - Volume 1: NLPCS (ICEIS 2017), eds Sharp B. Zock M. (Funchal: SciTePress), 228–237.

[B64] McRae K. Hare M. Ferretti T. R. Elman J. L. (2001). Activating verbs from typical agents, patients, instruments, and locations via event schemas, in Proceedings of the Twenty-Third Annual Conference of the Cognitive Science Society, eds Moore J. D. Stenning K. (Edinburgh: Cognitive Science Society), 617–622.

[B65] Mirković J. Altmann G. T. M. (2019). Unfolding meaning in context: the dynamics of conceptual similarity. Cognition 183, 19–43. 10.1016/j.cognition.2018.10.018 30408707

[B66] Moscovitch M. Cabeza R. Winocur G. Nadel L. (2016). Episodic memory and beyond: the hippocampus and neocortex in transformation. Annu. Rev. Psychol. 67, 105–134. 10.1146/annurev-psych-113011-143733 26726963 PMC5060006

[B67] Piai V. Anderson K. L. Lin J. J. Dewar C. Parvizi J. Dronkers N. F.. (2016). Direct brain recordings reveal hippocampal rhythm underpinnings of language processing. Proc. Natl. Acad. Sci. U.S.A. 113, 11366–11371. 10.1073/pnas.1603312113 27647880 PMC5056038

[B68] Poria S. Cambria E. Hazarika D. Mazumder N. Zadeh A. Morency L.-P. (2017). Context-dependent sentiment analysis in user-generated videos, in Proceedings of the 55th Annual Meeting of the Association for Computational Linguistics (Volume 1: Long Papers), eds Barzilay R. Kan M.-Y. (Vancouver, BC: Association for Computational Linguistics), 873–883.

[B69] Rubio-Fernández P. (2016). How redundant are redundant color adjectives? An efficiency-based analysis of color overspecification. Front. Psychol. 7:153. 10.3389/fpsyg.2016.00153 26924999 PMC4760116

[B70] Schlangen D. Baumann T. Atterer M. (2009). Incremental reference resolution: the task, metrics for evaluation, and a Bayesian filtering model that is sensitive to disfluencies, in Proceedings of the SIGDIAL 2009 Conference: The 10th Annual Meeting of the Special Interest Group on Discourse and Dialogue, eds Healey P. Pieraccini R. Byron D. Young S. Purver M. (London: Association for Computational Linguistics), 30–37.

[B71] Schlangen D. Zarrieß S. Kennington C. (2016). Resolving references to objects in photographs using the words-as-classifiers model, in Proceedings of the 54th Annual Meeting of the Association for Computational Linguistics (Volume 1: Long Papers), eds Erk K. Smith N. A. (Berlin: Association for Computational Linguistics), 1213–1223.

[B72] Shekhar R. Pezzelle S. Klimovich Y. Herbelot A. Nabi M. Sangineto E. (2017). FOIL it! Find one mismatch between image and language caption, in Proceedings of the 55th Annual Meeting of the Association for Computational Linguistics (Volume 1: Long Papers), eds Barzilay R. Kan M.-Y. (Vancouver, BC: Association for Computational Linguistics), 255–265.

[B73] Snedeker J. Yuan S. (2008). Effects of prosodic and lexical constraints on parsing in young children (and adults). J. Mem. Lang. 58, 574–608. 10.1016/j.jml.2007.08.001 19190721 PMC2390868

[B74] Spivey M. J. Huette S. (2013). Chapter 1: Toward a situated view of language, in Visually Situated Language Comprehension, Vol. 93 of *Advances in Consciousness Research*, eds Knoeferle P. Pyykkönen-Klauck P. Crocker M. W. (Amsterdam; Philadelphia, PA: John Benjamins Publishing Company), 1–52.

[B75] Spivey-Knowlton M. Sedivy J. C. (1995). Resolving attachment ambiguities with multiple constraints. Cognition 55, 227–267. 10.1016/0010-0277(94)00647-4 7634760

[B76] Suhr A. Lewis M. Yeh J. Artzi Y. (2017). A corpus of natural language for visual reasoning, in Proceedings of the 55th Annual Meeting of the Association for Computational Linguistics (Volume 2: Short Papers), eds Barzilay R. Kan M.-Y. (Vancouver, BC: Association for Computational Linguistics), 217–223.

[B77] Tan H. Bansal M. (2018). Object ordering with bidirectional matchings for visual reasoning, in Proceedings of the 2018 Conference of the North American Chapter of the Association for Computational Linguistics: Human Language Technologies, Volume 2 (Short Papers), eds Walker M. Ji H. Stent A. (New Orleans, LO: Association for Computational Linguistics), 444–451.

[B78] Tanenhaus M. K. Spivey-Knowlton M. J. Eberhard K. M. Sedivy J. C. (1995). Integration of visual and linguistic information in spoken language comprehension. Science 268, 1632–1634. 10.1126/science.7777863 7777863

[B79] Townsend D. J. Bever T. G. (2001). Sentence Comprehension: The Integration of Habits and Rules, A Bradford Book. Cambridge, MA: MIT Press.

[B80] Trueswell J. C. Tanenhaus M. K. Kello C. (1993). Verb-specific constraints in sentence processing: separating effects of lexical preference from garden-paths. J. Exp. Psychol. Learn. Mem. Cogn. 19, 528–553. 10.1037/0278-7393.19.3.528 8501429

[B81] Van Berkum J. J. A. Brown C. M. Zwitserlood P. Kooijman V. Hagoort P. (2005). Anticipating upcoming words in discourse: evidence from ERPs and reading times. J. Exp. Psychol. Learn. Mem. Cogn. 31, 443–467. 10.1037/0278-7393.31.3.443 15910130

[B82] Wang C. Niepert M. Li H. (2018). LRMM: learning to recommend with missing modalities, in Proceedings of the 2018 Conference on Empirical Methods in Natural Language Processing, eds Riloff E. Chiang D. Hockenmaier J. Tsujii J. (Brussels: Association for Computational Linguistics), 3360–3370.

[B83] Weber A. Braun B. Crocker M. W. (2006). Finding referents in time: eye-tracking evidence for the role of contrastive accents. Lang. Speech 49, 367–392. 10.1177/00238309060490030301 17225671

[B84] Witten I. B. Knudsen E. I. (2005). Why seeing is believing: merging auditory and visual worlds. Neuron 48, 489–496. 10.1016/j.neuron.2005.10.020 16269365

[B85] Wolfe J. M. (2012). When do I quit? The search termination problem in visual search, in The Influence of Attention, Learning, and Motivation on Visual Search, Vol. 59 of *Nebraska Symposium on Motivation*, eds Dodd M. D. Flowers J. H. (New York, NY: Springer), 183–208. 10.1007/978-1-4614-4794-8_8 PMC3979292 23437634

[B86] Yao Y. Xu J. Wang F. Xu B. (2018). Cascaded mutual modulation for visual reasoning, in Proceedings of the 2018 Conference on Empirical Methods in Natural Language Processing, eds Riloff E. Chiang D. Hockenmaier J. Tsujii J. (Brussels: Association for Computational Linguistics), 975–980.

[B87] Yu J. Jiang J. (2019). Adapting BERT for target-oriented multimodal sentiment classification, in Proceedings of the Twenty-Eighth International Joint Conference on Artificial Intelligence, IJCAI-19, ed Kraus S. (Macao: International Joint Conferences on Artificial Intelligence Organization), 5408–5414.

[B88] Zadeh A. Chen M. Poria S. Cambria E. Morency L.-P. (2017). Tensor fusion network for multimodal sentiment analysis, in Proceedings of the 2017 Conference on Empirical Methods in Natural Language Processing, eds Palmer M. Hwa R. Riedel S. (Copenhagen: Association for Computational Linguistics), 1103–1114.

[B89] Zadeh A. Zellers R. Pincus E. Morency L.-P. (2016). Multimodal sentiment intensity analysis in videos: facial gestures and verbal messages. IEEE Intell. Syst. 31, 82–88. 10.1109/MIS.2016.94

[B90] Zarrieß S. Schlangen D. (2017a). Is this a child, a girl or a car? Exploring the contribution of distributional similarity to learning referential word meanings, in Proceedings of the 15th Conference of the European Chapter of the Association for Computational Linguistics: Volume 2, Short Papers, eds Lapata M. Blunsom P. Koller A. (Valencia: Association for Computational Linguistics), 86–91.

[B91] Zarrieß S. Schlangen D. (2017b). Obtaining referential word meanings from visual and distributional information: experiments on object naming, in Proceedings of the 55th Annual Meeting of the Association for Computational Linguistics (Volume 1: Long Papers), eds Barzilay R. Kan M.-Y. (Vancouver,BC: Association for Computational Linguistics), 243–254.

[B92] Zhang Q. Fu J. Liu X. Huang X. (2018). Adaptive co-attention network for named entity recognition in tweets, in The Thirty-Second AAAI Conference on Artificial Intelligence (New Orleans, LO: AAAI Press), 5674–5681.

